# Disparities of Perceptions and Practices Related to Cervical Cancer Prevention and the Acceptability of HPV Vaccination According to Educational Level in a French Cross-Sectional Survey of 18–65 Years Old Women

**DOI:** 10.1371/journal.pone.0109320

**Published:** 2014-10-10

**Authors:** Julie Haesebaert, Delphine Lutringer-Magnin, Julie Kalecinski, Giovanna Barone, Anne-Carole Jacquard, Yann Leocmach, Véronique Régnier, Philippe Vanhems, Franck Chauvin, Christine Lasset

**Affiliations:** 1 Université Lyon 1, CNRS UMR 5558 et Centre Léon Bérard, Lyon, France; 2 Institut de Cancérologie de la Loire et Université Jean Monnet de Saint-Étienne, IFR 143, Saint-Étienne, France; 3 Sanofi-Pasteur MSD, Lyon, France; 4 Université Lyon 1, CNRS UMR 5558 et Hospices Civils de Lyon, Lyon, France; Old Dominion University, United States of America

## Abstract

**Introduction:**

We aimed to study the relationships between educational level, women's knowledge about cervical cancer (CC), and acceptance of HPV vaccination for their daughters.

**Methods:**

We analysed data from a quantitative (self-administrated questionnaire) and qualitative (semi-structured interviews) cross-sectional study performed in 2008 among 1,229 French 18–65-year-old women recruited by general practitioners. Women were categorized into three educational level groups: low (LEL: 43.9%), medium (MEL: 33.4%) and high (HEL: 22.6%).

**Results:**

Knowledge about CC and its prevention was lower among LEL women. In the 180 mothers of 14–18-year-old daughters (99 LEL, 54 MEL, 45 HEL), acceptance of HPV vaccine was higher in LEL (60.4%) and MEL (68.6%) than in HEL mothers (46.8%). Among LEL mothers, those who were favourable to HPV vaccination were more likely to be young (OR = 8.44 [2.10–34.00]), to be vaccinated against hepatitis B (OR = 4.59 [1.14–18.52]), to have vaccinated their children against pneumococcus (OR = 3.52 [0.99–12.48]) and to present a history of abnormal Pap smear (OR = 6.71 [0.70–64.01]).

**Conclusion:**

Although LEL women had poorer knowledge about CC and its prevention, they were more likely to accept HPV vaccination than HEL mothers.

## Background

A total of 2,800 cases of cervical cancer (CC) leading to 1,000 deaths occurred in France in 2011 [1]. Most of these cases were potentially avoidable. Complementing the Papanicolaou smear (PS) screening test, vaccination against *Human Papillomavirus* (HPV) provides a new opportunity to decrease the CC burden. Since 2007 in France, the HPV vaccine has been recommended for all girls and young women aged 14 and aged 15–23 years who are within the first year of sexual activity [2]. PS is recommended every 3 years for women aged 25–65 years old [3]. In 2007–09, PS coverage, ie women who had had a PS within the past three years was estimated at 58.5% [3]. Women of low socioeconomic status are less likely to have had PS, and are more prone to CC [4], [5]. HPV vaccination could potentially have the greatest benefit in this population. However, there is concern that uptake of the vaccine, just as with the uptake of screening, may be lower in this group than in the wider population, so perpetuating existing disparities in risk.

Mothers play a crucial role in the uptake of vaccination, and it is therefore important to assess factors associated with their intention to vaccinate their daughters, especially in underserved populations. Knowledge of the link between CC and HPV is very poor among the less well educated [6]. Limiting factors include the fear of side effects, concern that HPV immunization might promote more risky sexual behaviour [7]–[10], and, particularly in underserved communities, the cost of the vaccine [8], [11], [12].

Most studies on HPV vaccination perceptions in underserved population have been performed in developing countries, in ethnic minorities or in the United States [13]. With the exception of United Kingdom, little research on CC and HPV awareness has been carried out in Europe [13]. The French context is particularly interesting in several factors. First, the recommended age for immunization is old compared with that in other countries (14 years and above). Secondly, a controversy emerged in the 1990s over a supposed link between the hepatitis B vaccination of adolescents and multiple sclerosis. This brought a halt to the immunization campaign. This particular scare about vaccination may have had long-term effects on the perceived safety of mass vaccination in France. Finally, the 65% to 100% reimbursement of HPV vaccine costs by the national social and the additional private health insurances could provide a favourable setting, particularly among underserved populations.

Our objectives were 1) to assess knowledge about CC, PS and the HPV vaccine among 18–65 year-old women in relation to socio-economic status measured by educational level; and 2) to investigate determinants of HPV vaccine acceptance among mothers of low educational level. The latter investigation was focused on women with daughters aged 14–18, an age range chosen to encompass girls above the age at which vaccination is recommended in France and below the age at which they can themselves legally assent to the procedure.

This study was part of the REMPAR (Recherche et Evaluation des Moyens de Prévention Anti-HPV en Rhône-Alpes) programme aimed at evaluating means of preventing HPV-mediated disease.

## Methods

### Study design and population

This study is based on the data collected for the HPV-FEM study [14], a cross sectional survey conducted in June and July 2008 among women aged 18–65 living in the Rhône-Alpes (RA) region (10% of the French population). Participants were recruited by 39 general practitioners (GPs) during consecutive consultations, independently of the reason for consultation, with no exclusion criteria. The participating GPs were from a representative sample of 279 GPs in the RA region recruited for a prior study of the REMPAR program [15].

### Evaluation of socio-economic status

Socioeconomic status was defined by educational level. It has been shown that women with low educational status have a higher cancer and CC incidence and mortality rate [16], [17]. Based on the International Standard Classification of Education (ISCED) [18], women were categorized into 3 groups: low educational level, LEL (ISCED level 0-1-2: pre-primary, primary, lower secondary); medium - MEL (ISCED level 3–4: upper secondary, post-secondary non tertiary); and high - HEL (ISCED 5–6: first or second stage of tertiary education).

### Data collection

The HPV-FEM study used two interrelated methodologies: quantitative, by self-administered, anonymous questionnaire, and qualitative, with face to face semi-structured interviews. Women who agreed to participate were asked to complete the questionnaire after their GP consultation and before they left the practice premises. The questionnaire has been described elsewhere in detail [14] and is available as Figure S1. Briefly, it consisted of multiple choice and open-ended questions covering five areas: 1) socio-demographic data; 2) practices relating to disease prevention; 3) gynaecological history; 4) knowledge about CC and its prevention; and 5) attitude towards HPV vaccination for their daughters. Acceptability of the HPV vaccine was assessed by response to six questions (Table 1) on the basis of which women were classified as favourable, uncertain or opposed. The understandability of the questionnaire had been established through a pilot study involving three focus groups of 12 women each (from low, medium and high educational level). It was also tested in underserved women recruited from two centres offering free health care. Qualitative data were obtained from face to face, semi-structured interviews conducted by a sociologist with underserved LEL mothers of 14–18 year old daughters. These women had all completed the self-administered questionnaire. They were identified by GPs working in underserved areas (defined according to mean household income of the zip-code) or by medical centres specifically designated for the underserved population. These women were offered a voluntary 30–60 minutes interview at home or at the social centre. The interview grid covered in greater depth items of the questionnaire with emphasis on the drivers of and barriers to acceptance of HPV vaccination. Interview transcripts were compiled and imported into the qualitative software NVivo (QSR International) for analysis. The analysis grid was designed to build on the topics addressed in the quantitative part of the study.

**Table 1:** Acceptability of HPV vaccination: options presented in the self-administered questionnaire, and the coding of responses.

About vaccination to prevent cervical cancer, if you have a daughter: Positive response coded as	
1. I will get some information and consider it	*Uncertain*
2. I prefer to wait	*Uncertain*
3. She(they) is(are) already vaccinated	*Favourable*
4. I intend to vaccinate my daughter(s) in the future	*Favourable*
5. I will vaccinate my daughter(s) if she(they) asks me	*Uncertain*
6. I think that this vaccination is useless	*Opposed*

### Statistical analysis

We compared the characteristics and knowledge of women according to educational level and analyzed factors related to the acceptability of the HPV vaccine among LEL mothers of a 14–18 year old daughter: women favourable to vaccination were compared against those who were unfavourable (ie uncertain or opposed) (Table 1). Categorical variables were compared using the Chi-square or Fisher's exact tests and continuous variables using Student's or Mann-Whitney tests. Results with a p-value ≤ 0.05 were considered statistically significant. In relation to determinants of vaccine acceptability, backward descending logistic regression was used to discriminate the most suitable model for multivariate analysis. Variables that achieved a p-value ≤ 0.2 on univariate analysis were entered in the model. Two models were tested with two threshold outputs: 5% and 10%. Data analysis was performed using SAS 9.1 software (SAS Institute, Cary, NC).

### Ethics

This study was approved by the French National Committee for personal data protection in medical research. Each included women was given oral information and a written information notice by her GP. In accordance with the French regulatory on research participation, a verbal consent was obtained by the GP for women who filled the questionnaire and a written consent was obtained for women who participated to interviews with the sociologist.

## Results

### Population

On the 1,478 women included, 100 (6.8%) were students at the time of the study and 149 (10.1%) did not provide information on their educational level. As a result, data from 1,229 women were analyzed, of whom 540 (43.9%) were categorized as LEL, 411 (33.4%) MEL and 278 (22.6%) HEL. A total of 188 (15.3%) were mothers of at least one 14–18 year-old daughter: 99 (8.1%) of LEL, 54 (4.4%) of MEL and 35 (2.8%) of HEL. Eighteen of the LEL mothers of a 14–18 year old daughter were interviewed.

The socio-demographic characteristics, attitudes towards prevention and gynaecological history of participants are presented in Table 2. Women with LEL were older than those in other educational categories (mean age: 44.2 years for LEL, 39.0 for MEL and 39.1 for HEL, p<0.001). Frequency of usual gynaecological follow-up and of adequate PS screening increased from LEL to HEL (p = 0.004 for follow-up and p<0.001 for PS). The PS of LEL women was more frequently performed by a GP (29.4%) as opposed to a gynaecologist than was the case with MEL (20.1%) and HEL (4.2%) women (p<0.001). In the interviews, LEL mothers said they complied with their physician's recommendation if PS was suggested.

**Table 2:** Sociodemographic characteristics and preventive health behaviours of survey participants according to educational level.

Characteristic	LEL (N = 540)	MEL (N = 411)	HEL (N = 278)	p value
**Age** (years)				
18–29	74 (13.7)	89 (21.7)	37 (13.3)	<0.001
30–39	106 (19.6)	135 (32.9)	114 (41.0)	
40–49	175 (32.4)	113 (27.5)	89 (32.0)	
50–65	185 (34.3)	74 (18.0)	38 (13.7)	
**Employment**				
Employed	321 (62.8)	316 (81.0)	225 (85.9)	<0.001
Jobless/housewife/retired	190 (37.2)	74 (19.0)	37 (14.1)	
***Missing data***	*29*	*21*	*16*	
**Current or last employment**				
Farmer	7 (1.5)	7 (1.8)	2 (0.8)	<0.001
Tradesman	26 (5.4)	20 (5.2)	11 (4.4)	
Executive	7 (1.5)	26 (6.7)	107 (42.8)	
Foreman	14 (2.9)	35 (9.0)	25 (10.0)	
Employee	366 (75.8)	281 (72.8)	105 (42.0)	
Worker	63 (13.0)	17 (4.4)	0 (0.0)	
***Missing data***	*57*	*25*	*28*	
**Social/financial assistance** 1	42 (8.2)	12 (3.0)	5 (1.8)	<0.001
**Family situation**				
Married or living with partner	383 (72.0)	323 (78.8)	219 (78.8)	0.023
Single/Divorced/Widowed	149 (28.0)	87 (21.2)	59 (21.2)	
***Missing data***	*8*	*1*	*0*	
**Vaccine status themselves**				
DT-IPV and BCG vaccine	495 (91.7)	393 (95.6)	267 (96.0)	0.060
Measles, Mumps and Rubella	232 (42.9)	208 (50.6)	130 (46.8)	<0.001
Hepatitis B	223 (41.3)	236 (57.4)	178 (64.0)	<0.001
**Vaccine status of their children** 2				
DT-IPV and BCG vaccine	447 (95.7)	318 (97.6)	212 (96.8)	0.372
Measles, Mumps and Rubella	316 (67.7)	265 (81.3)	185 (84.5)	0.001
Chickenpox	57 (12.2)	32 (9.8)	6 (2.7)	<0.001
Pneumococcus	90 (19.3)	82 (25.2)	59 (26.9)	0.040
Hepatitis B	256 (60.7)	153 (49.0)	101 (48.1)	0.001
**Current cigarette smoker**	116 (23.1)	62 (16.2)	24 (9.5)	<0.001
**Usual frequency of gynaecologic check-up**				
Each year	303 (59.3)	278 (68.1)	198 (72.0)	0.004
Every 2–3 years	123 (24.1)	85 (20.8)	57 (20.7)	
Less frequently/never	85 (16.6)	45 (11.0)	20 (7.3)	
***Missing data***	*29*	*3*	*3*	
**Last Pap test within the past 3 years**	418 (77.4)	353 (85.9)	247 (88.8)	<0.001
**History of abnormal Pap test**	44 (8.1)	54 (13.1)	41 (14.7)	0.018
**History of gynaecologic surgery**	71 (13.1)	43 (10.4)	28 (10.1)	0.220
**History of sexually transmitted infection**	17 (3.1)	36 (8.8)	26 (9.4)	<0.001

LEL: Low education level, MEL: Medium education level, HEL: High education level, DTP: Diphtheria-tetanus-poliomyelitis.

1 Beneficiary of public free health insurance and/or minimal financial allocation for non workers.

2 N = 1,012 mothers: 467 LEL, 326 MEL and 219 HEL.

### Knowledge about CC and its prevention

LEL women had significantly poorer knowledge about PS screening (Table 3). In interviews, LEL mothers did not fully understand the role of PS screening, explaining for example that PS is *“to check for abnormalities*” but without mentioning CC. In relation to the cause of CC, 15.7% of LEL, 33.1% of MEL and 47.8% of HEL women responded with “HPV” or a closely related answer (“viral infection” or “STIs”) (p<0.001). LEL women were significantly less likely than more educated groups to have heard of the HPV vaccine and to know the target population and the recommended age of immunization. In interviews, only 2 (of 18) LEL mothers were able to mention HPV as the cause of CC. Even though 6 knew that the CC vaccine targeted an STI, they had not made the link between HPV and CC.

**Table 3:** Knowledge about cervical cancer, Pap-smear screening and HPV vaccination.

Question	LEL (N = 540)	MEL (N = 411)	HEL (N = 278)	p value
**What is the role of the Pap test?**				
To prevent cervical cancer	301 (**55.7**)	269 (**65.4**)	201 (**72.3**)	**<0.001**
Other response (no information given or incorrect answer, eg to treat cervical cancer, to prevent all gynaecologic cancers, to monitor ovaries)	239 (44.3)	142 (34.6)	77 (27.7)	
**When should women have a Pap test?**				
During the whole of adult life	438 (81.1)	351 (85.4)	235 (84.5)	0.176
Other response (no information or incorrect answer, eg only before or after menopause)	102 (18.9)	60 (14.6)	43 (15.5)	
**How frequently should women have the Pap test?**				
Every 2–3 years (French national recommendations)	222 (41.1)	187 (45.5)	105 (37.8)	**<0.001**
Yearly	277 (51.3)	215 (52.3)	167 (60.1)	
Other response (no information or incorrect response, eg once or from time to time)	41 (7.6)	9 (2.2)	6 (2.2)	
**What is the cause of cervical cancer?**				
HPV	43 (8.0)	93 (22.6)	87 (31.3)	**<0.001**
Related response (eg an STI, viral infection)	37 (6.9)	41 (10.0)	43 (15.5)	
Incorrect or no response	460 (85.2)	277 (67.4)	148 (53.2)	
**Have you heard of HPV vaccination?**	370 (68.5)	340 (82.7)	235 (84.5)	**<0.001**
**Who should be vaccinated?**				
Young girls before first intercourse (or within a year of first intercourse) or similar answer	167 (30.9)	175 (42.6)	122 (43.9)	**<0.001**
Incorrect/no answer	373 (69.0)	236 (57.4)	156 (56.1)	
**What is the recommended age for vaccination?**				
14 –23 years or similar answer	266 (49.3)	254 (61.8)	168 (60.4)	**<0.001**
Incorrect/no answer	274 (50.7)	157 (38.2)	110 (39.6)	

LEL: Low education level, MEL: Medium education level, HEL: High education level.

### HPV vaccine acceptance and reasons given by mothers

A total of 188 women were mothers of a 14–18 year old daughter. Among the 174 mothers who gave an opinion, 60.3% were favourable towards the vaccine and 47.6% of them had already had their daughters vaccinated. Interestingly, HEL mothers had the lowest acceptance rate (46.9%) vs. 68.6% for MEL and 60.4% for LEL (Table 4). Among the 18 LEL mothers interviewed, 13 were favourable towards the vaccine. Whatever the level of education, the main reason given by favourable mothers was the opportunity to prevent their daughters from developing a severe disease (mentioned by 72.7% of favourable LEL mothers, 65.7% of MEL and 47.0% of HEL). In interviews, LEL mothers often referred to their “*fear of cancer*”. The majority of mothers' decisions to vaccinate were based on their physicians' opinion: “*I can trust him*”, was a typical response. Whatever their level of education, the main reason given by unfavourably disposed mothers was the newness of the vaccine or the fear of side effects (70.6% of unfavourable HEL mothers, 56.2% of MEL and 41.7% of LEL), as evoked during interviews: “*Sometimes vaccines induce diseases. I’m afraid because this vaccine is new*”. As an example, LEL interviewees mentioned their belief that the hepatitis B vaccine could cause multiple sclerosis, reflecting the French controversy. Other barriers related to the young age of their daughters: 4 mothers (2 LEL and 2 HEL) thought their daughters were too young to talk about sexual issues. The cost of the vaccine was mentioned by one HEL mother. In interviews, some LEL mothers explained that, for religious reasons, their daughters should have no sex before marriage and so should not be at risk of infection. They thought HPV vaccination was not a priority as their daughters were too young to become sexually active and therefore preferred to wait. Another barrier specifically raised by three LEL mothers interviewed was the fear that HPV immunization could affect their daughters' fertility.

**Table 4:** HPV vaccine acceptance among mothers of 14–18 year old daughters (N = 188).

Position	LEL (N = 99)	MEL (N = 54)	HEL (N = 35)	p value
**Favourable**	**55 (60.4)**	**35 (68.6)**	**15 (46.9)**	0.143
My daughter(s) is/are already vaccinated	23 (25.3)	20 (39.2)	7 (21.9)	
I intend to vaccinate my daughter(s) in the future	32 (35.1)	15 (29.4)	8 (25.0)	
**Uncertain/Opposed**	**36 (39.56)**	**16 (31.4)**	**17 (53.1)**	
I will get some information and consider it	18 (19.8)	10 (19.6)	8 (25.0)	
I prefer to wait	12 (13.2)	1 (2.0)	8 (25.0)	
I will vaccinate my daughter(s) if she(they) asks me	5 (5.5)	5 (9.8)	1 (3.1)	
I think that this vaccination is useless (Opposed)	1 (1.1)	0 (0.0)	0 (0.0)	
**Missing data**	*8*	*3*	*3*	

LEL: Low education level, MEL: Medium education level, HEL: High education level.

### Factors independently associated with vaccine acceptance among LEL mothers

Among 99 LEL mothers, 91 (91.9%) gave their opinion on HPV vaccination: 55 (60.4%) were favourable while 36 (39.6%) were uncertain/opposed. Table 5 presents the results of the univariate analysis of factors associated with HPV vaccine acceptance. No significant association between vaccine acceptability and practice of PS, knowledge about CC and PS was identified. After multivariate analysis, only younger age remained associated with a favourable opinion towards HPV vaccination (OR: 6.29 (1.71–23.1)) (Table 6). In the model with the 10% output threshold, the mother's own hepatitis B vaccination status remained also significant (OR: 4.59 (1.14–18.5)). Having had her child vaccinated against *pneumococcus* (OR: 3.52 (0.99–12.48)) and history of abnormal PS (OR: 6.71 (0.70–64.01)) were borderline (Table 6).

**Table 5:** Univariate analysis of factors associated with HPV vaccination acceptance among mothers with a low educational level (N = 91).

	Favourable (N = 55)	Uncertain/opposed (N = 36)	Crude OR (95% CI)	p value
Age <40 years old *vs* ≥ 40 years old	20 (36.4)/35 (63.6)	3 (8.3)/33 (91.7)	6.29 (1.71–23.14)	0.003
Gainful employment *vs* no gainful employment	40 (74.1)/15 (25.9)	27 (77.1)/9(22.9)	0.89 (0.34–2.32)	0.743
Being beneficiary of social/financial assistance *vs* not beneficiary	4 (7.7)/51 (92.3)	1 (2.9)/35 (97.1)	2.75 (0.29–25.7)	0.357
Married or living with partner *vs* single/divorced/widow	41 (78.9)/14 (21.1)	32 (88.9)/4 (11.1)	2.15 (0.63–7.37)	0.218
Hepatitis B immunization status (themselves)				
Did not remember/missing data *vs* no	14 (25.5)/20 (36.4)	4 (11.1)/22 (61.1)	3.85 (1.09–13.65)	0.037
Yes *vs* No	21 (38.2)/20 (36.4)	10 (27.8)/22 (61.1)	2.31 (0.88–6.07)	0.090
Pneumococcal immunization (children) Yes *vs* No*	15 (27.3)/40 (72.7)	5 (13.9)/31 (86.1)	2.33 (0.76–7.09)	0.132
Usual gynecologic check-up frequency: Each year or every 2–3 years *vs* less frequently	44 (81.5)/11 (18.5)	31 (86.1)/5 (13.9)	0.57 (0.16–1.98)	0.369
History of gynaecologic surgery *vs* no history of gynaecologic surgery	4 (7.8)/51 (92.2)	1 (3.0)/35 (97.0)	2.75 (0.29–25.66)	0.363
History of STI *vs* no history of STI	2 (4.1)/53 (95.9)	3 (10.7)/52 (89.3)	0.42 (0.07–2.65)	0.347
Last Pap test within last 3 years *vs* older than 3 years	45 (81.8)/10 (18.2)	29 (80.6)/7(19.4)	1.36 (0.45–4.15)	0.591
History of abnormal Pap test result *vs* no history of abnormal Pap test result	8 (14.8)/47 (85.2)	1 (3.0)/35 (97.0)	5.96 (0.71–49.85)	0.083
Knowing Pap test should be performed during the whole of adult life *vs* incorrect or no response	43 (78.2)/12 (21.8)	31 (86.1)/5 (13.9)	0.58 (0.19–1.82)	0.343
Knowing Pap test frequency according to French national recommendations *vs* incorrect or no response	21 (38.2)/34 (71.8)	17 (47.2)/19 (52.8)	0.69 (0.29–1.62)	0.393
Knowing Pap test is to prevent cervical cancer *vs* incorrect or no response	31 (56.4)/24 (43.6)	24 (66.7)/12 (43.3)	0.65 (0.27–1.56)	0.326
Knowing cervical cancer is due to HPV or STI *vs* incorrect or no response	11 (20.0)/44 (80.0)	8 (22.2)/28 (77.8)	0.88 (0.32–2.45)	0.799
Knowing who should be vaccinated against HPV according to recommendations *vs* incorrect or no response	27 (49.1)/28 (50.9)	14 (38.9)/22 (61.1)	1.52 (0.65–3.57)	0.339
Knowing the recommended age for HPV vaccination *vs* incorrect or no response	31 (56.4)/14 (43.6)	22 (61.1)/14 (48.9)	0.82 (0.35–1.93)	0.653

Results are presented as N (%)/N(%) for number and percent for the tested category versus number and percent for the reference category.

OR: Odds ratio, CI: confidence interval, vs: versus, STI, Sexually transmitted infection.

**Table 6:** Multivariate analysis of factors associated with HPV vaccination acceptance among mothers with a low educational level (N = 91).

	Model 1†		Model 2‡	
	Adjusted OR (95% CI)	p value	Adjusted OR (95% CI)	p value
Age				
≥ 40 years old	1		1	
<40 years old	6.29 (1.71–23.14)	0.006	8.44 (2.10–34.00)	0.003
Hepatitis B immunization status (themselves)				
No	NS		1	
Did not remember/missing data			2.18 (0.74–6.47)	0.160
Yes			4.59 (1.14–18.52)	0.032
Pneumococcal immunization (children)				
No	NS		1	
Yes			3.52 (0.99–12.48)	0.051
History of abnormal Pap test result				
No	NS		1	
Yes			6.71 (0.70–64.01)	0.098

† : first model, output threshold = 0.05, ‡: second model, output threshold = 0.10, *OR: Odd ratio, CI: confidence interval, NS not significant*.

## Discussion

Our results revealed disparities in CC prevention knowledge and practice across educational levels. Importantly, although LEL women had poorer knowledge about CC, PS screening and HPV immunization than women with more education, they were more likely than HEL mothers to accept HPV vaccination.

Knowledge of CC and HPV was poor, especially in LEL women: 8% of LEL women were able to mention HPV as the cause of CC. Interviews among LEL women highlighted that they were unaware of the relationship between PS, HPV and CC [6], [9], [19], [20]. Even so, HPV immunization was well accepted: 60.3% of all mothers of 14–18 year-old daughters said they intended to or already had vaccinated their daughters. Though the difference was not statistically significant, acceptance of HPV vaccine seemed to vary across educational level: over 60% of LEL and MEL mothers were favourable, compared to 47% of HEL mothers. Previous studies found a higher acceptance rate among poorly educated parents for HPV vaccination [7], [11], [21], [22]. Nevertheless, the intention to vaccinate needs to be turned into actual vaccination [23]. The difficulty in providing vaccination for underserved girls is not in convincing them or their mothers but in improving their access to the healthcare system [24].

Whatever their educational level, mothers justified their acceptance of the vaccine mainly in terms of wanting to keep their daughters healthy and protecting them against this anxiogenic and potentially fatal disease [25], [26]. Fear of side effects is the major barrier to vaccination [11], [27], [28]. LEL mothers revealed some misconceptions such as the fear that their daughter could have difficulties in becoming pregnant because of the vaccination. These misconceptions were probably linked to poor understanding of body and vaccine functions [29]. Religious belief was another important factor, leading to the belief that vaccination could wait since their daughters should not have sex before marriage. Reduced acceptance of vaccines has been found among people who regularly practiced a religion [7], [12], [30]. However, fear that the vaccine would encourage their daughters to engage in earlier or more risky sexual activity was not clearly evident in our study [9], [11], [31]. This may reflect cultural differences, or the fact that the recommended age for HPV vaccination in France is higher than in other countries.

Factors associated with HPV vaccine acceptance among LEL mothers in our study were a younger age and their opinion of vaccination in general. In contrast to previous studies [9], [32], [33], no link was found between acceptance in LEL mothers and frequency of gynaecological or PS follow-up. This could be explained by a lack of power, but also by the poor understanding among LEL mothers of the link between PS and HPV vaccination. The cost of the vaccine was not cited by LEL mothers as a barrier to vaccination even though it is an important concern raised in the literature [11], [12]. The explanation may be due to the French reimbursement of HPV vaccination reaching 100% of costs for the most underserved women on social assistance (8% of our LEL sample), thanks to the French social health insurance providing a social health care program for families and individuals with low income and resources. For others a 65% reimbursement is proposed by the national health insurance, which is usually completed by the private additional insurance to 100%.

There were certain limitations in our study. Firstly, due to recruitment during a GPs consultation, our sample is drawn from women with access to healthcare. Secondly, the use of a self-administered questionnaire could to some extent bias our LEL population by excluding the least educated women who could not read or write in French. Thirdly, the identification of underserved women was only based on educational level. However this criterion is one of the most widely used socio-economic indicators; and its association with cancer incidence and mortality (including CC) and with PS uptake is established [4], [17]. Finally, our relatively small sample of poorly educated women may have restricted our ability to identify determinants of HPV vaccination in this population, but the combination of qualitative and quantitative data allowed us to explore in greater detail women's knowledge and beliefs about HPV vaccination.

Although LEL mothers are less compliant with gynaecological follow-up and PS screening and have more limited knowledge about CC and its prevention, their acceptance of HPV vaccination is good. However, further information on vaccine is required to avoid misconceptions among LEL mothers and to improve its acceptance by HEL mothers. The major limitation to extending HPV immunization among the daughters of LEL mothers' may not be attitudes and beliefs but their restricted access to health care which prevents the translation of intentions into action.

## Supporting Information

Figure S1 **HPVFEM Questionnaire.** The questionnaire was given by the general practitioner to the included women. It was a self-administered questionnaire, anonymously filed by the women.
(PDF)
