# Supplementary material for: Disparities of Perceptions and Practices Related to Cervical Cancer Prevention and the Acceptability of HPV Vaccination According to Educational Level in a French Cross-Sectional Survey of 18–65 Years Old Women
Source: PLoS One. 2014 Oct 10;9(10):e109320. doi: 10.1371/journal.pone.0109320 (PMC4193774; doi:10.1371/journal.pone.0109320)
Supplement: Figure S1 — HPVFEM Questionnaire. The questionnaire was given by the general practitioner to the included women. It was a self-administered questionnaire, anonymously filed by the women. (PDF) [file pone.0109320.s001.pdf]

FEMMES

# QUESTIONNAIRE

PROGRAMME **REMPAR**

- A quelle date remplissez-vous ce questionnaire ?....... |__|__|/|__|__|/|__|__|__|__|
- Quel est votre âge ?.................................... |__|__| ans
- Quel est le code postal de votre domicile ?......... |__|__|__|__|__|
- Quel est votre état civil ? ..............................

| Mariée Vie maritale | Célibataire | Divorcée | Veuve |
|---|---|---|---|
| ☐ | ☐ | ☐ | ☐ |

- Quelle est votre situation professionnelle actuelle ? ☞ *Cochez une seule réponse*

| Salariée Trav. indépendante | Lycéenne Etudiante | Au chômage | Sans profession Mère au foyer | Retraitée | En invalidité |
|---|---|---|---|---|---|
| ☐ | ☐ | ☐ | ☐ | ☐ | ☐ |

- Quel est ou quel a été votre dernier emploi ?

| Agriculteur | Artisan Commerçant Chef d'entreprise | Cadre | Technicien Agent de maîtrise | Employé | Ouvrier |
|---|---|---|---|---|---|
| ☐ | ☐ | ☐ | ☐ | ☐ | ☐ |

- Si vous n'êtes pas lycéenne/étudiante, quand avez-vous arrêté vos études ?

| Arrêt avant 14 ans | Arrêt avant le Bac | Bac à Bac + 2 | Bac + 3 à Bac + 4 | Bac + 5 et plus |
|---|---|---|---|---|
| ☐ | ☐ | ☐ | ☐ | ☐ |

- Etes-vous bénéficiaire : ...............................

| de la CMU | du RMI | Aucun des 2 |
|---|---|---|
| ☐ | ☐ | ☐ |

## *Vous, vos enfants et la vaccination*

- Avez-vous eu les vaccins suivants ? ☞ *Cochez une seule réponse pour chaque vaccin*

| Vaccins obligatoires (BCG - DTPolio) | Rougeole - Oreillons - Rubéole (ROR) | Hépatite B |
|---|---|---|
| ☐ Oui ☐ Non ☐ Je ne sais plus | ☐ Oui ☐ Non ☐ Je ne sais plus | ☐ Oui ☐ Non ☐ Je ne sais plus |

- Avez-vous des enfants ? ...............................

| Oui | Non |
|---|---|
| ☐ | ☐ |

➲ SI OUI :

- Décrivez votre famille :

| | Enfant ① | Enfant ② | Enfant ③ | Enfant ④ | Enfant ⑤ | Enfant ⑥ | Enfant ⑦ | Enfant ⑧ |
|---|---|---|---|---|---|---|---|---|
| Sexe<br>☞ *Ecrivez F pour Fille, G pour Garçon* | | | | | | | | |
| Age | | | | | | | | |

- D'une manière générale, quels vaccins vos enfants ont-ils eu ? ☞ *Cochez les vaccins réalisés*

| Vaccins obligatoires (BCG - DTPolio) | Rougeole Oreillons Rubéole | Pneumocoque | Papillomavirus | Varicelle | Rotavirus (gastro-entérite) |
|---|---|---|---|---|---|
| ☐ | ☐ | ☐ | ☐ | ☐ | ☐ |

- Ont-ils été vaccinés contre l'hépatite B ? ..... Tous ☐ Certains ☐ Aucun ☐

  - Ceux qui ont été vaccinés, l'ont-ils été plutôt : Avant 5 ans ☐ A l'adolescence/ A l'âge adulte ☐

## Vous et votre suivi médical

- Suivez-vous actuellement un traitement ? .......... Oui ☐ Je vais le commencer ☐ Non ☐

➲ SI OUI :

- Lequel ? ______________________________

NE RIEN INSCRIRE DANS CES CASES

- Pour quelle raison ? ______________________________

NE RIEN INSCRIRE DANS CES CASES

- Avez-vous eu des mammographies dans le cadre du dépistage du cancer du sein ? ☞ *Cochez une seule réponse*

| Oui, avec prescription de mon médecin | Oui, à la suite d'un courrier reçu pour un dépistage gratuit | Non | Pas encore |
|---|---|---|---|
| ☐ | ☐ | ☐ | ☐ |

- Avez-vous eu une aide à l'arrêt du tabac ? ☞ *Si vous n'avez jamais fumé, cochez la case correspondante*

| **Je n'ai jamais fumé** | Non car j'ai arrêté de moi-même | Oui et j'ai arrêté de fumer | Oui mais je n'ai pas arrêté de fumer | Non pas pour l'instant | Non mais je ne souhaite pas arrêter |
|---|---|---|---|---|---|
| ☐ | ☐ | ☐ | ☐ | ☐ | ☐ |

## Vous et votre suivi gynécologique

- Avez-vous déjà eu les interventions gynécologiques suivantes ? Laser du col de l'utérus ☐ Conisation du col de l'utérus ☐ Ablation totale de l'utérus ☐ Aucune des 3 ☐

☞ *Cochez la ou les cases correspondantes*

➲ SI OUI :

• Pour quelle(s) raison(s) ? ______________________________

______________________________

NE RIEN INSCRIRE DANS CES CASES

• En quelle(s) année(s) ? .......................... |_|_|_|_| |_|_|_|_|

▪ Avez-vous eu un traitement par médicaments pour une maladie gynécologique transmissible ?... Oui ☐ Non ☐

➲ SI OUI :

• Pour quelle(s) maladie(s) ? ______________________________

______________________________

NE RIEN INSCRIRE DANS CES CASES

▪ Quelle méthode de contraception avez-vous déjà utilisée ? ☞ *Cochez une ou plusieurs réponses*

| Je n'ai jamais utilisé de contraception | Préservatifs | Pilule contraceptive | Stérilet | Autres méthodes |
|---|---|---|---|---|
| ☐ | ☐ | ☐ | ☐ | ☐ ______ |
| | | Age de début |_|_| | | |

▪ En dehors de tout problème de santé, avez-vous un suivi gynécologique ? ☞ *Cochez une seule réponse*

| Au moins 1 fois par an | Tous les 2-3 ans | Rarement | Jamais |
|---|---|---|---|
| ☐ | ☐ | ☐ | ☐ |

▪ Quand avez-vous fait pour la dernière fois un frottis ? ☞ *Cochez une seule réponse*

| Il y a environ 1 an | Entre 2 à 3 ans | Il y a plus de 3 ans | Je n'en ai jamais fait | Je ne sais pas/plus |
|---|---|---|---|---|
| ☐ | ☐ | ☐ | ☐ | ☐ |

▪ Quel médecin a fait ce dernier frottis ? ☞ *Cochez une seule réponse* ............................................................ Généraliste ☐ Gynécologue ☐ Autre ☐ ______________

▪ Avez-vous déjà eu un frottis anormal ? ☞ *Cochez une seule réponse*

| Oui, une fois | Oui, plus d'une fois | Non | Je ne sais pas/plus |
|---|---|---|---|
| ☐ | ☐ | ☐ | ☐ |

➲ SI OUI :

• En quelle année la première fois ? ............. |_|_|_|_|

• Avez-vous été re-contactée par votre médecin après un frottis anormal ?............................... Oui ☐ Non ☐

➲ SI OUI :

• Pourquoi ? ______________________________

NE RIEN INSCRIRE DANS CES CASES

## *Les informations que vous avez sur le cancer du col de l'utérus et ses moyens de prévention*

▪ Le rôle du frottis est : *☞ Cochez une seule réponse*

| De surveiller les ovaires et le col de l'utérus | De prévenir tous les cancers gynécologiques | De prévenir le cancer du col de l'utérus | Je n'ai pas d'informations |
|---|---|---|---|
| ☐ | ☐ | ☐ | ☐ |

▪ Il faut faire un frottis : *☞ Cochez une seule réponse*

| Tous les ans | Tous les 2-3 ans | De temps en temps | Une fois et ça suffit | Je n'ai pas d'informations |
|---|---|---|---|---|
| ☐ | ☐ | ☐ | ☐ | ☐ |

▪ Il faut faire un frottis : *☞ Cochez une seule réponse*

| Toute la vie adulte (> 20 ans) | Seulement jusqu'à la ménopause | Seulement après la ménopause | Je n'ai pas d'informations |
|---|---|---|---|
| ☐ | ☐ | ☐ | ☐ |

▪ Avez-vous entendu parler de la cause du cancer du col de l'utérus ? ......................... Oui ☐ Non ☐

➲ SI OUI :

• Quelle est sa cause ? ____________________

NE RIEN INSCRIRE DANS CES CASES

└┘└┘└┘ └┘└┘└┘ └┘└┘└┘

▪ Avez-vous entendu parler du vaccin contre le cancer du col de l'utérus ? ................. Oui ☐ Non ☐

➲ SI OUI :

• Quand la première fois ?......................... mois └┘└┘ année └┘└┘└┘└┘

• Comment avez-vous eu ces informations ? *☞ Cochez une ou plusieurs réponses*

| Par mon médecin | Par un autre professionnel de santé (pharmacien, etc.) | Par mon entourage | A la télévision | A la radio<br>Dans la presse écrite<br>Sur Internet | Autre |
|---|---|---|---|---|---|
| ☐ | ☐ | ☐ | ☐ | ☐ | ☐ ________ |

Et avec ces informations, vous pouvez dire :

• Qui est concerné par ce vaccin : ____________________

____________________

NE RIEN INSCRIRE DANS CES CASES

└┘└┘└┘ └┘└┘└┘ └┘└┘└┘

• A quel âge il est conseillé de se faire vacciner : ____________________

____________________

NE RIEN INSCRIRE DANS CES CASES

└┘└┘└┘ └┘└┘└┘ └┘└┘└┘

• Qui doit faire des frottis après la vaccination : ____________________

____________________

| NE RIEN INSCRIRE DANS CES CASES | | |
|---|---|---|
| ⊔⊔⊔ | ⊔⊔⊔ | ⊔⊔⊔ |

# *A propos de cette vaccination contre le cancer du col de l'utérus*

- Pour votre fille (vos filles), si vous en avez, quel que soit son(leur) âge : ☞ *Cochez une seule réponse*

| Je vais m'informer et y réfléchir | Je préfère attendre | Elle est (elles sont) déjà vaccinée(s) | J'ai décidé de faire vacciner ma fille (mes filles) | Je vaccinerai ma fille (mes filles) si elle(s) me le demande(nt) | Je pense que cette vaccination est inutile |
|---|---|---|---|---|---|
| ☐ | ☐ | ☐ | ☐ | ☐ | ☐ |

➲ QUELLE QUE SOIT VOTRE REPONSE :

- Dîtes pourquoi : ____________________________________________

______________________________________________________________

______________________________________________________________

______________________________________________________________

| NE RIEN INSCRIRE DANS CES CASES | | |
|---|---|---|
| ⊔⊔⊔ | ⊔⊔⊔ | ⊔⊔⊔ |

Une fois renseigné, glissez votre questionnaire dans l'enveloppe.

Remettez-la à la secrétaire de votre médecin ou dans la boîte du cabinet médical.

Nous vous remercions de votre participation à cette étude

qui contribuera à faire progresser la prise en charge du cancer du col de l'utérus.

Si vous souhaitez des informations, vous pouvez nous contactez au 04.78.78.27.45.
